# A population-based epidemiology of Wilson’s disease in South Korea between 2010 and 2016

**DOI:** 10.1038/s41598-020-70976-1

**Published:** 2020-08-20

**Authors:** Eun Ju Choe, Jong Won Choi, Minjin Kang, Yong Kang Lee, Han Ho Jeon, Byung Kyu Park, Sun Young Won, Yong Suk Cho, Jeong Hun Seo, Chun Kyon Lee, Jae Bock Chung

**Affiliations:** 1 Department of Internal Medicine, National Health Insurance Service Ilsan Hospital, Goyang, Korea; 2 Research Institute, National Health Insurance Service Ilsan Hospital, Goyang, Korea

**Keywords:** Medical research, Gastroenterology, Hepatology

## Abstract

Very few population-based studies have examined the epidemiology of Wilson’s disease (WD). We investigated the epidemiology of WD using the National Health Insurance Service (NHIS) database in South Korea. We analyzed not only the statistical variables of WD, but also those of WD-related diseases. WD patients were identified with the relevant International Classification of Diseases-10 code out of 50.5 million people. We used the NHIS database from 2009 to 2016 and analyzed the incidence rate, prevalence, and clinical symptoms of WD. A total of 1,333 patients were identified. The average annual incidence rate was 3.8 per million person-years. The prevalence was 38.7 per million people. The mean diagnostic age was 26.1 ± 17.2 with earlier diagnosis in men (*P* = 0.0003). Among the patients, 988 (74.1%) had hepatic symptoms, 510 (38.3%) had neurologic symptoms, and 601 (45.1%) had psychiatric symptoms. Before the diagnosis of WD, 350 (26.3%) had neurologic symptoms, and 427 (32%) had psychiatric symptoms. The annual mortality rate was 0.7%. Age, liver cirrhosis, and liver failure correlated with a fatal prognosis (*P* < 0.05). Many patients showed neurologic and psychiatric symptoms before they were diagnosed with WD. Prognosis correlated with age, liver cirrhosis, and liver failure.

## Introduction

Wilson’s disease (WD) is an autosomal-recessive disorder associated with copper metabolism that produces abnormal accumulation of copper in the liver, brain, kidneys, and other organs^1^. It is caused by mutations in the *ATP7B* gene, which is involved in transporting copper across cell membranes^2,3^. Genetic variation among mutations leads to a broad spectrum of clinical manifestations, including hepatic, neurologic, and psychiatric symptoms^4^.

Generally, patients are diagnosed with WD after experiencing hepatic symptoms. When patients present without specific symptoms or the initial symptoms are neurologic or psychiatric, the diagnosis of WD can be delayed. However, neuropsychiatric symptoms are pretty common, occurring in about a third of WD patients^5^. Studies have reported that neurologic and psychiatric manifestations are present in up to 40% and 10–25% of patients respectively, at the time they are diagnosed with WD^6,7^. Thus, research into neuropsychiatric aspects of WD is currently receiving attention.

Most patients with WD are diagnosed before they are 40 years old, although WD has been reported at ages from 2 to 80 years^8–10^. In the past, the incidence rate of WD was reported to be 30 per million person-years^1^. But a recent study in Hong Kong reported an average annual incidence rate of 1.44 per million person-years, and a recent study in France found a prevalence of 15 per million person-years^11,12^. Only a few epidemiological studies about WD have been carried out, and most of them are more than 30 years old. Changes in the diagnostic criteria, including gene analysis and quantitative serologic study, enabled by the progression of diagnostic technology have created a need for new epidemiological studies of WD. Thus, our aim in this study was to evaluate the incidence rate, prevalence, treatment efficacy, and symptoms of WD in South Korea using the National Health Insurance Service (NHIS) database.

## Methods

### Data source and population

We used the NHIS database from 2009 to 2016 to identify patients with WD in South Korea. All residents, approximately 50.5 million people, must be enrolled in the health insurance provided by the NHIS, and all types of medical institutions must register with the NHIS. The NHIS maintains a database containing complete details about all the participants and their diagnoses, medical treatments, and deaths. Therefore, it is an excellent source of epidemiological data.

WD patients were defined as people who had an outpatient visit or admission history with the principal diagnostic code of E830 in the Korean Standard Classification of Diseases 2015 (KCD), which is the Korean version of the International Classification of Disease, 10th Revision. Due to the possibility of miscoding, we also used data from the special system of registration for rare and intractable diseases for medical insurance in South Korea. To evaluate the incidence rate of WD, we excluded WD patients from 2009 to avoid including previously diagnosed cases and analyzed only patients who were diagnosed with WD between 2010 and 2016.

This study was approved by the Institutional Review Board of NHIS Ilsan Hospital, South Korea (IRB No. 2018-01-012). To guarantee the confidentiality of the data and records, we adhered to the Ethical Principles for Human Research defined by the 1964 Declaration of Helsinki, revised and updated by the World Health Organization (Fortaleza 2000). All the data used in this study were routinely collected for the NHIS database. Therefore informed consent was not required.

### Diagnosis of WD

Any diagnosis of WD must be confirmed by clinical, biochemical, and molecular genetic tests in patients with two or more of the following symptoms: Kayser-Fleischer rings, low serum ceruloplasmin, high 24-h urinary copper excretion, and high accumulation of unbound copper in the liver. Because WD requires a complex diagnostic process with specific clinical parameters, KCD code E830 is unlikely to be used in error. In this study, the WD diagnosis date is taken to be the first date that KCD code E830 was registered. The diagnosis dates for associated diseases are also taken to be the first date of each disease code registration.

### Associated disease

We identified and analyzed the Electronic Data Interchange (EDI) codes in the NHIS database for each of the following diseases in patients with WD (KCD E830). Hepatic presentations were chronic hepatitis, acute hepatitis, fulminant hepatitis, liver cirrhosis, and hepatocellular carcinoma (HCC). Neurologic presentations were Parkinsonism, tremor, dystonia, ataxia, chorea, dysarthria, dementia, and seizures. Psychiatric presentations were depression, manic disorder, bipolar disorder, anxiety disorder, cognitive impairment, personality disorder, sleep disturbances, schizophrenia, somatoform disorder, and tic disorder. Renal presentations include Fanconi’s syndrome, renal tubular acidosis, hematuria, proteinuria, and hypercalcemia. Cardiac presentations were cardiomyopathy and arrhythmia. The endocrine system manifestation was hypoparathyroidism. Osteoarticular manifestations were arthralgia, osteoarthritis, osteomalacia, and osteoporosis.

### Treatment methods

For the period following each WD patient, we reviewed their medical treatment and liver transplantation histories. Generally, penicillamine, zinc acetate hydrate, and trientine are used to treat WD. We analyzed the prescribing history of each medication using EDI codes and examined the clinical course of all the WD patients.

### Outcomes and statistical analysis

Patients were included from the time they were diagnosed with WD and followed up during the whole study period or until death. Newly diagnosed cases of WD were defined upon a patient’s first registration of KCD code E830. We calculated the incidence and prevalence of WD during the entire study period. The prevalence of WD was defined as the number of individuals visiting medical institutions who were assigned KCD code E830 for WD as a primary diagnosis among national residents’ registration data of NHIS. The incidence rate of WD was also analyzed. We calculated incidence as age-standardized rates and crude rates per million person-years using 2010 national residents' registration data. A chi-squared test was used to analyze the differences between the incidence rates of WD according to age groups. The prevalence and incidence rates were also analyzed according to the disease categories associated with WD. The ratio of men and women and the age at the diagnosis were also analyzed according to the disease categories associated with WD. The mortality of WD was calculated by dividing the number of deaths by the total number of WD patients. The annual mortality of WD patients by sex and age were also analyzed. We carried out survival analysis using Cox proportional hazards models to identify the statistical significance of the categories, such as age, sex, and treatment modalities. The risk of all-cause mortality during the follow-up period after a diagnosis of WD was analyzed as factors correlated with a fatal prognosis. To verify significance and survival rate of a liver transplantation, survival curves for that were plotted using the Kaplan–Meier method. The data were described using the frequency and percentage of the total for categorical variables and the mean and standard deviation for continuous variables. Independent t-testing was used for continuous variables. Statistical analyses were performed using SAS Studio 3.4, Enterprise Edition (SAS Institute Inc., Cary, NC, USA) on a remote-control system for assessing the NHIS database.

## Results

### Incidence rates of WD

**Figure 1:**
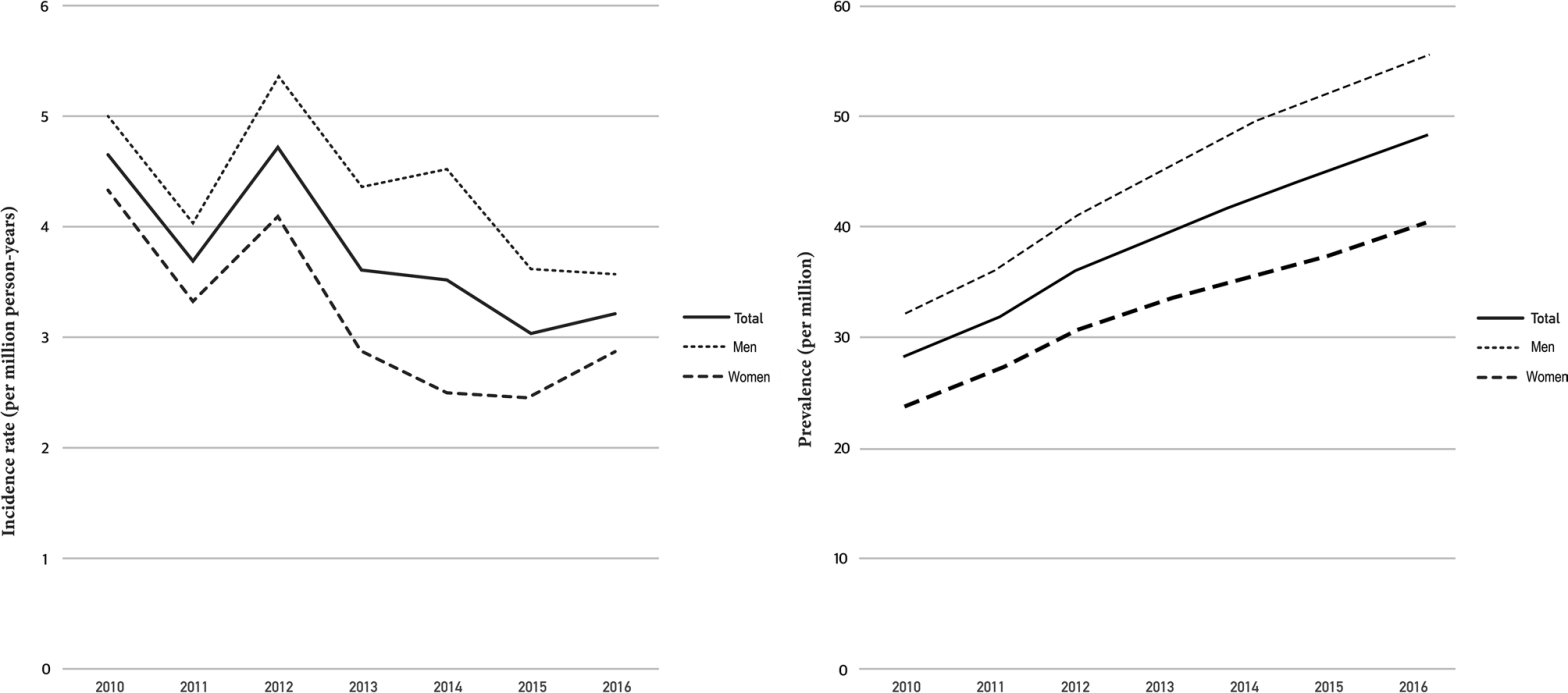
Annual incidence and prevalence rates of Wilson’s disease by sex.

During the study period, 1,333 patients (768 (57.6%) men and 565 (42.4%) women) were diagnosed with WD out of 50.5 million people enrolled on the health insurance provided by the NHIS. The average annual incidence rate was 3.8 per million person-years (Fig. 1). The mean diagnostic age was 26 ± 17.2, 24.6 ± 15.3 in men and 28 ± 19.3 in women, with earlier diagnosis in men (*P* = 0.0003). The incidence of WD was 7.1 per million person-years in the under 19 age group, 5.7 in the 20–29 age group, 3.2 in the 30–39 age group, 2.2 in the 40–49 age group, 2.2 in the 50–59 age group, 1.2 in the 60–69 age group, 0.9 in the 70–79 age group, and 0.5 in the older than 80 group. Most patients (77.8%) were diagnosed before they were 40 years old with statistical significance (Table 1). The difference in annual incidence rates between patients under 40 and the patients over 40 was statistically significant (*P* = 0.0001). There was no statistically significance of the incidence rates of WD between sexes. The annual average incidence rates were similar (Table 1).

**Table 1:** Annual incidence rates and prevalence of Wilson’s disease.

	Population	2010	2011	2012	2013	2014	2015	2016	Total
Incidence rates (per million person-years)									
New patients	50,515,666	235 (4.7)	186 (3.7)	238 (4.7)	182 (3.6)	177 (3.5)	153 (3)	162 (3.2)	1,333 (3.8)
Sex									
Men	25,310,385	126 (5)	102 (4)	135 (5)	110 (4.3)	114 (4.5)	91 (3.6)	90 (3.6)	768 (4.3)
Women	25,205,281	109 (4.3)	84 (3.3)	103 (4.1)	72 (2.9)	63 (2.5)	62 (2.5)	72 (2.9)	565 (3.2)
Age (years)									
0–19	11,606,790	89 (7.7)	75 (6.5)	84 (7.2)	80 (6.9)	88 (7.6)	76 (6.5)	82 (7.1)	574 (7.1)
20–29	6,866,956	53 (7.7)	40 (5.8)	53 (7.7)	40 (5.8)	32 (4.7)	27 (3.9)	28 (4.1)	273 (5.7)
30–39	8,370,549	46 (5.5)	27 (3.2)	32 (3.8)	24 (2.9)	21 (2.5)	20 (2.4)	20 (2.4)	190 (3.2)
40–49	8,844,352	30 (3.4)	22 (2.5)	34 (3.8)	15 (1.7)	16 (1.8)	6 (0.7)	12 (1.4)	135 (2.2)
50–59	7,066,823	11 (1.6)	12 (1.7)	26 (3.7)	13 (1.8)	13 (1.8)	17 (2.4)	15 (2.1)	107 (2.2)
60–69	4,191,329	3 (0.7)	5 (1.2)	6 (1.4)	6 (1.4)	6 (1.4)	6 (1.4)	3 (0.7)	35 (1.2)
70–79	2,618,525	3 (1.1)	3 (1.1)	3 (1.1)	3 (1.1)	1 (0.4)	1 (0.4)	2 (0.8)	16 (0.9)
≥ 80	950,342	0 (0)	2 (2.1)	0 (0)	1 (1.1)	0 (0)	0 (0)	0 (0)	3 (0.5)
Prevalence rates (per million people)									
Total patients	50,515,666	1,415 (28)	1589 (31.5)	1817 (36)	1984 (39.3)	2,149 (42.5)	2,285 (45.2)	2,430 (48.1)	13,669 (38.7)
Sex									
Men	25,310,385	814 (32.2)	909 (35.9)	1,040 (41.1)	1,140 (45)	1,247 (49.3)	1,329 (52.5)	1,405 (55.5)	7,884 (44.5)
Women	25,205,281	601 (23.8)	680 (27)	777 (30.8)	844 (33.5)	902 (35.8)	956 (37.9)	1,025 (40.7)	5,785 (32.8)
Age (years)									
0–19	11,606,790	513 (44.2)	587 (50.6)	669 (57.6)	745 (64.2)	831 (71.6)	907 (78.1)	989 (85.2)	5,241 (64.5)
20–29	6,866,956	405 (59)	442 (64.4)	495 (72.1)	534 (77.8)	565 (82.3)	591 (86.1)	615 (89.6)	3,647 (75.9)
30–39	8,370,549	300 (35.8)	324 (38.7)	353 (42.2)	373 (44.6)	393 (47)	407 (48.6)	422 (50.4)	2,572 (43.9)
40–49	8,844,352	126 (14.2)	148 (16.7)	179 (20.2)	192 (21.7)	205 (23.2)	209 (23.6)	219 (24.8)	1,278 (20.6)
50–59	7,066,823	47 (6.7)	56 (7.9)	82 (11.6)	93 (13.2)	105 (14.9)	118 (16.7)	131 (18.5)	632 (12.8)
60–69	4,191,329	14 (3.3)	18 (4.3)	23 (5.5)	27 (6.4)	31 (7.4)	34 (8.1)	36 (8.6)	183 (6.2)
70–79	2,618,525	8 (3.1)	11 (4.2)	13 (5)	16 (6.1)	16 (6.1)	17 (6.5)	16 (6.1)	97 (5.3)
≥ 80	950,342	2 (2.1)	3 (3.2)	3 (3.2)	4 (4.2)	3 (3.2)	2 (2.1)	2 (2.1)	19 (2.9)

^†^2010 year standard population.

### Prevalence of WD

**Figure 2:**
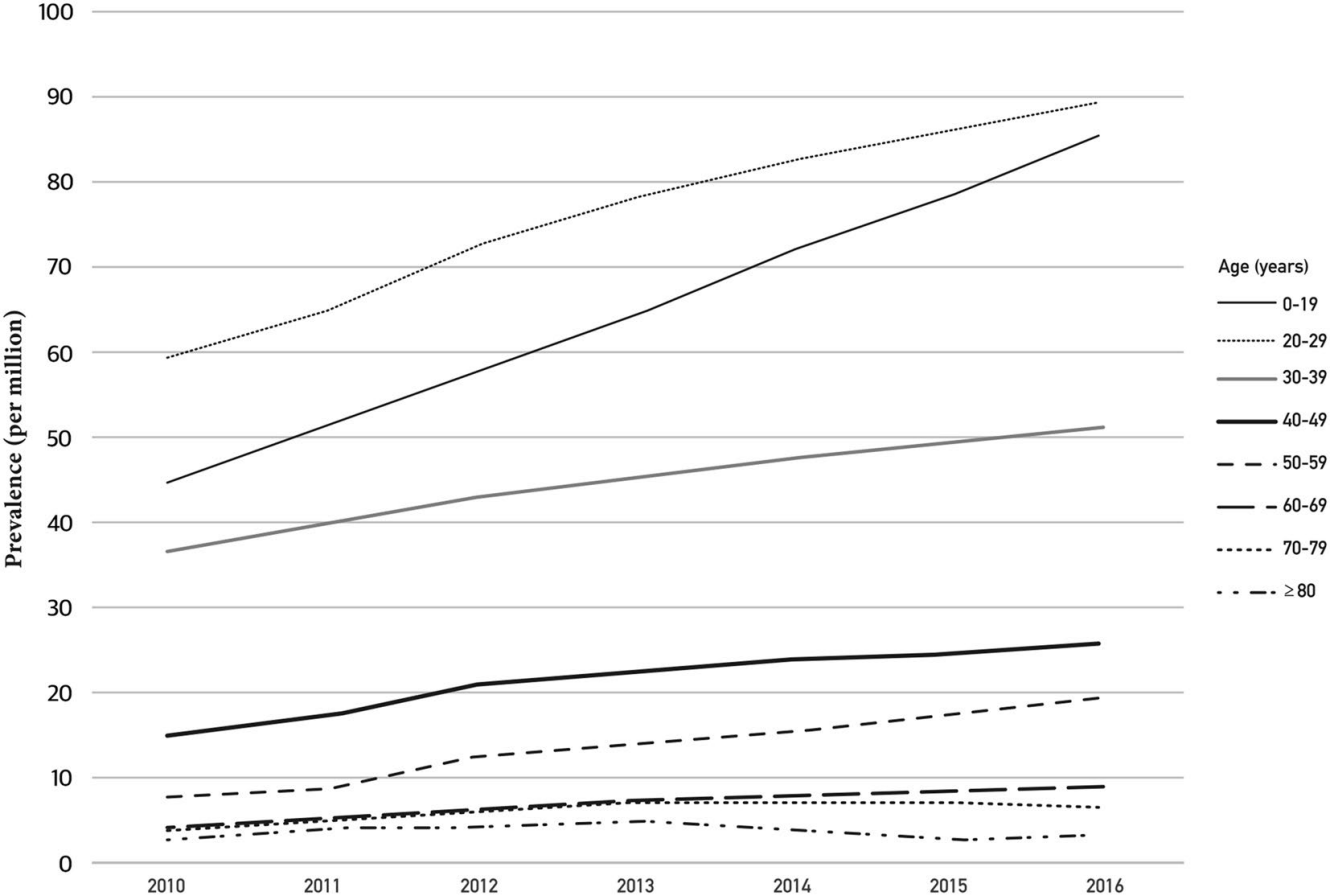
Annual prevalence rates of Wilson’s disease by age.

The prevalence of WD was 38.7 per million people, with 13,669 cumulative patients. The prevalence of WD increased annually in both sexes, 28 per million people in 2010, 31.5 in 2011, 36 in 2012, 39.3 in 2013, 42.5 in 2014, 45.2 in 2015, and 48.1 in 2016 (Table 1). The prevalence of WD was 44.5 per million people among men and 32.8 per million people among women, which was a statistically insignificant difference (Fig. 1). The prevalence of WD was 64.5 per million people in the under 19 age group, 75.9 in the 20–29 age group, 43.9 in the 30–39 age group, 20.6 in the 40–49 age group, 12.8 in the 50–59 age group, 6.2 in the 60–69 age group, 5.3 in the 70–79 age group, and 2.9 in the older than 80 age group. Among all patients, 83.4% were younger than 40. The prevalence peaked among people in their 20 s, and the next highest age group was teens. Although the prevalence of WD increased in all age groups, the tendency was marked in groups younger than 40 and slight in the groups older than 70 (Fig. 2).

### Diseases associated with WD

#### Liver disease

Among all WD patients, 74.1% had liver disease. The mean age at the diagnosis of liver disease was 27 ± 17.3, similar to the mean age at the diagnosis of WD (26 ± 17.2). The mean age at the diagnosis of liver disease was 25.1 ± 15.5 in men and 29.4 ± 19.2 in women; men were significantly younger (*P* = 0.0001). The most common type of hepatic complication was hepatitis, which was reported in 68.6% of WD patients, and 21.8% of patients presented with liver failure. The mean onset age of fulminant hepatitis was 32 ± 16.5, which is older than the mean age at the diagnosis of WD. Liver cirrhosis was observed in 17.3% of patients, ascites in 6.8%, esophageal varices in 3.6%, gastric varices in 1%, and hepatorenal syndrome in 1.1%. HCC occurred in 9.9% of all WD patients, with a mean diagnostic age of 33.7 ± 17 (Table 2). Fifty-five of the 132 WD patients with HCC (41.7%) were diagnosed with HCC before receiving a WD diagnosis.

**Table 2:** Proportion of patients with hepatic, neurologic, and psychiatric symptoms.

Valuables	Patients, N (%) N = 1,333	Men (%)	Age (year)
Liver disease	988 (74.1)	560 (56.7)	27 ± 17.3
Hepatitis	915 (68.6)	520 (56.8)	27.3 ± 17.5
Fulminant hepatitis	291 (21.8)	156 (53.6)	32 ± 16.5
Liver cirrhosis	230 (17.3)	125 (54.3)	33.2 ± 16.9
HCC	132 (9.9)	65 (49.2)	33.7 ± 17
Ascites	91 (6.8)	39 (42.8)	34.1 ± 16.9
Esophageal varices	48 (3.6)	31 (64.6)	37.7 ± 14.6
Gastric varices	13 (1.0)	8 (61.5)	31.9 ± 14.5
Hepatorenal syndrome	15 (1.1)	6 (40.0)	40.8 ± 17.2
Neurologic disease	510 (38.3)	320 (62.7)	31.5 ± 16.6
Parkinsonism	155 (11.6)	97 (62.6)	38.3 ± 14.1
Tremor	186 (14.0)	129 (69.4)	32.2 ± 14
Dystonia	186 (14.0)	102 (54.8)	28.6 ± 17.4
Ataxia	158 (11.9)	95 (60.1)	35.5 ± 15.4
Chorea	17 (1.3)	10 (58.8)	35.1 ± 14.3
Dysarthria	15 (1.1)	8 (53.3)	31.9 ± 21
Dementia	56 (4.2)	38 (67.9)	42.4 ± 15.9
Seizure	219 (16.4)	136 (62.1)	32.3 ± 16.9
Psychiatric disease	601 (45.1)	339 (56.4)	32.6 ± 16.3
Depression	333 (25.0)	181 (54.4)	35.5 ± 15.9
Manic disorder	7 (0.5)	3 (42.9)	25.7 ± 13.3
Bipolar disorder	118 (8.9)	66 (55.9)	34.6 ± 16.3
Anxiety disorder	405 (30.4)	229 (56.5)	34.7 ± 16.1
Cognitive impairment	49 (3.7)	28 (57.1)	18.7 ± 14.8
Personality disorder	54 (4.1)	35 (64.8)	25.9 ± 14.4
Sleep disturbance	273 (20.5)	137 (50.2)	38.2 ± 14.8
Schizophrenia	116 (8.7)	65 (56.0)	35.3 ± 15.84
Somatoform disorder	18 (1.4)	6 (33.3)	42.6 ± 14.3
Tic disorder	20 (1.5)	15 (75.0)	21.4 ± 13.3

HCC, hepatocellular carcinoma.

#### Neurologic disease

Neurologic diseases were observed in 38.3% of all WD patients. The ratio of men to women was 62.7%. The mean age of symptom onset was 31.5 ± 16.6, 29.2 ± 15.1 in men and 35.2 ± 18.2 in women, indicating a significantly earlier onset in men (*P* < 0.0001). Neurologic symptoms were prevalent in the order of seizures 16.4%, tremor 14%, dystonia 14%, ataxia 11.9%, Parkinsonism 11.6%, dementia 4.2%, chorea 1.3%, and dysarthria 1.1%. The mean age at the diagnosis of neurologic diseases was older than that of WD (Table 2). But 350 of the 510 patients with neurologic disease (68.6%) had a history of treatment for neurologic symptoms before they were diagnosed with WD. Before the diagnosis of WD, 58.8% of chorea patients had already been treated, 56.5% of tremor patients, 46.8% of dystonia patients, 44.8% of seizure patients, 43.2% of parkinsonism patients, 39.9% of ataxia patients, 28.6% of dementia patients, and 20% of dysarthria patients. Therefore, 350 WD patients (26.3%) were treated for neurologic symptoms without WD being considered as a causative factor.

#### Psychiatric disease

Psychiatric disease was observed in 45.1% of all WD patients, making it the second most common manifestation following liver disease. The ratio of men to women was 56.4%. Symptoms first presented at age 32.6 ± 16.3 on average, 29.5 ± 14.7 in men and 36.6 ± 17.3 in women. Symptom onset age was significantly earlier in men (*P* < 0.0001). Psychiatric symptoms occurred in the order of anxiety disorder 30.4%, depression 25%, sleep disturbance 20.5%, bipolar disorder 8.9%, schizophrenia 8.7%, personality disorder 4.1%, cognitive impairment 3.7%, tic disorder 1.5%, somatoform disorder 1.4%, and manic disorder 0.5% (Table 2). And 427 of the 601 patients with psychiatric disease (71%) had a history of treatment for psychiatric symptoms before they were diagnosed with WD. Before the diagnosis of WD, 75% of tic disorder patients had already been treated, 64.8% of personality disorder patients, 57.1% of cognitive impairment patients, 56.5% of anxiety disorder patients, 56% of schizophrenia patients, 55.9% of bipolar disorder patients, 54.4% of depression patients, 50.2% of sleep disturbance patients, 42.9% of manic disorder patients, and 33.3% of somatoform disorder patients. Therefore, 427 WD patients (32%) were treated for psychiatric symptoms without WD being considered as a causative factor.

#### Other diseases

Renal disease was observed in 4.4% of all WD patients. The ratio of men to women was 60.3%. The mean age at diagnosis of renal disease was 28 ± 17.6 years, and that of Fanconi’s syndrome was 29.2. Renal presentations occurred in the order of Fanconi’s syndrome 2%, renal tubular acidosis 0.9%, hypercalcemia 0.7%, hematuria 0.5%, and proteinuria 0.4%.

Cardiac disease was observed in 13% of all WD patients. The ratio of men to women was 54.9%. The mean age at diagnosis with cardiac disease was 35.6 ± 17.2 years, that of cardiomyopathy was 41.8 years, and that of arrhythmia was 35.9 years. Arrhythmia occurred in 13% and cardiomyopathy in 1.3% of patients.

Hypoparathyroidism was observed in 1.3% of all WD patients. The ratio of men to women was 47.1%, The mean age at diagnosis with hypoparathyroidism was 37.9 ± 14.6 years.

Osteoarticular disease was observed in 64.5% of all WD patients. The ratio of men to women was 57.6%. The mean age for osteoarticular disease was 30 ± 16.6 years. Osteoarticular presentations included arthralgia 60.7%, osteoarthritis 22.1%, osteoporosis 6.8%, and osteomalacia 5%.

### Treatment and progress

#### Mortality rates from WD

The annual mortality rate among WD patients was 0.7% on average. From 2010 to 2016, the annual mortality rates were 0.9%, 0.6%, 0.8%, 0.6%, 0.8%, 0.7%, and 0.8%, respectively (Table 3). The rates were 0.7% in men and 0.7% in women, which was an insignificant difference. The mortality rate among WD patients was 0.2% in the under 19 age group, 0.4% in the 20–29 age group, 0.9% in the 30–39 age group, 1.2% in the 40–49 age group, 2.5% in the 50–59 age group, 6.2% in the 60–69 age group, 4.9% in the 70–79 age group, and 14.3% in the older than 80 age group.

**Table 3:** Mortality rates (%) during follow-up among patients with Wilson’s disease.

Year (Patients)	2010 (N = 1,415)	2011 (N = 1589)	2012 (N = 1817)	2013 (N = 1984)	2014 (N = 2,149)	2015 (N = 2,285)	2016 (N = 2,430)	Total (N = 13,669)
Annual mortality, number (rate, %)								
No of fatal cases	12 (0.9)	10 (0.6)	15 (0.8)	12 (0.6)	17 (0.8)	17 (0.7)	19 (0.8)	102 (0.7)
Sex								
Men	7 (0.9)	4 (0.4)	10 (1.0)	7 (0.6)	9 (0.7)	14 (1.1)	7 (0.5)	58 (0.7)
Women	5 (0.8)	6 (0.9)	5 (0.6)	5 (0.6)	8 (0.9)	3 (0.3)	12 (1.2)	44 (0.7)
Age (years)								
0–19	1 (0.2)	2 (0.3)	4 (0.6)	2 (0.3)	0 (0)	0 (0.)	0 (0)	9 (0.2)
20–29	3 (0.7)	0 (0)	1 (0.2)	1 (0.2)	1 (0.2)	4 (0.7)	5 (0.8)	15 (0.4)
30–39	3 (1.0)	3 (0.9)	4 (1.1)	1 (0.3)	6 (1.5)	5 (1.2)	3 (0.7)	25 (0.9)
40–49	0 (0)	3 (2.0)	2 (1.1)	3 (1.6)	2 (1.0)	2 (1.0)	4 (1.8)	16 (1.2)
50–59	3 (6.4)	0 (0)	2 (2.4)	1 (1.1)	4 (3.8)	2 (1.7)	5 (3.8)	17 (2.5)
60–69	1 (7.1)	1 (5.6)	2 (8.7)	2 (7.4)	3 (9.7)	1 (2.9)	2 (5.6)	12 (6.2)
70–79	0 (0)	1 (9.1)	0 (0)	1 (6.3)	0 (0)	3 (17.7)	0 (0)	5 (4.9)
≥ 80	1 (50.0)	0 (0)	0 (0)	1 (25.0)	1 (33.3)	0 (0)	0 (0)	3 (14.3)

^†^The mortality rate was calculated by dividing the number of death cases in the corresponding year by the patients with Wilson’s disease from 2010 to 2016.

#### Prognosis and related factors

Fifty-two WD patients died during the follow-up period. Risk factors for mortality were analyzed (Table 4). Age was one of the risk factors (*P* < 0.0001), which is consistent with the mortality rate among WD patients, which also increased by age (Table 3). Aggravation of liver disease, including liver failure, and liver cirrhosis, was one of the risk factors related to mortality during follow-up. Medical treatment, conservative management, and liver transplantation did not showed significant correlation with mortality during follow-up (Table 4).

**Table 4:** Clinical characteristic of patients diagnosed with Wilson’s disease between 2010 and 2016.

Valuables	No of patients (%) Total = 1,333	No of death (%) Total = 52	Univariate analysis		*P* value
			HR	95%CI	
Age, years (mean SD)	26.1 ± 17.2	44.5 ± 19.3	1.00	0.00–0.00	< 0.0001
Men sex	768 (57.7)	31 (4.0)	1.09	0.63–1.91	0.753
Liver failure	38 (2.9)	9 (17.3)	6.55	3.18–13.46	< 0.0001
Liver cirrhosis	163 (12.2)	24 (46.2)	5.47	3.15–9.51	< 0.0001
Medical treatment	465 (34.9)	21 (40.4)	1.14	0.65–3.01	0.640
Penicillamine	124 (9.3)	14 (11.3)	1.00		
Penicillamine + zinc	21 (1.6)	0 (0.0)	0.00	0.00	0.997
Penicillamine + zinc + trientine	53 (4.0)	2 (3.8)	0.34	0.08–1.50	0.157
Penicillamine + trientine	45 (3.4)	4 (8.9)	0.84	0.28–2.58	0.764
Zinc	48 (3.6)	0 (0.0)	0.00	0.00	0.995
Zinc + trientine	98 (7.4)	0 (0.0)	0.00	0.00	0.993
Trientine	76 (5.7)	1 (1.3)	0.12	0.02–0.95	0.044
Liver transplantation	56 (4.2)	3 (5.4)	1.28	0.40–4.12	0.674
BCT	854 (64.1)	30 (57.7)	1.28	0.49–1.49	0.574

SD, standard deviation; BCT, best conservative treatment.

#### Medical treatment and liver transplantation

During study period, 465 WD patients received medical treatment (Table 4). Their overall mortality rate during follow-up was 4.5% (21 of 465). Among the medical treatment groups, the mortality rate was the highest in the penicillamine-alone group (11.3%, *P* > 0.05). The mortality rate during follow-up was 3.6% in patients who did not receive medical treatment. Table 4 shows factors correlated with prognosis in WD patients. Mortality during follow-up did not differ significantly with or without medical treatment.

**Figure 3:**
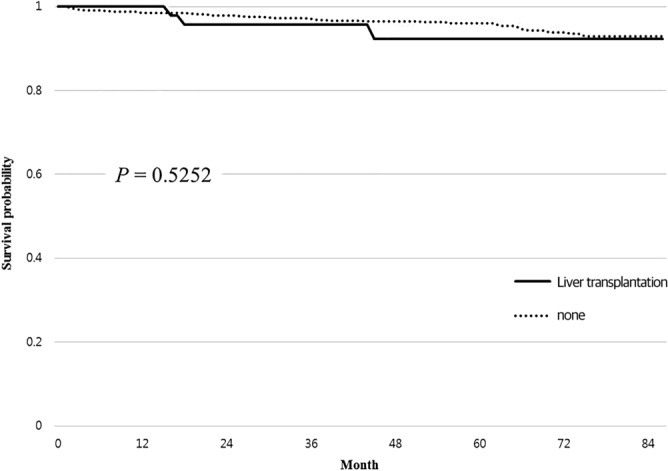
Kaplan–Meier curve for liver transplantation.

Fifty-six patients received liver transplants, 18 men and 38 women. The distribution of those patients by age was 34 in the under 19 age group (60.7%), 6 in the 20–29 age group (10.7%), 4 in the 30–39 age group (7.1%), 8 in the 40–49 age group (8.2%), and 4 in the 50–59 age group (7.1%). None of the transplant patients was older than 60. The mortality rate during follow-up among the liver transplant patients was 5.4% (3 of 56). The survival rate of patients who received a liver transplant was 100% at 1 year, 95.8% at 2 years, 95.8% at 3 years, and 92.3% at 4 years. However, receiving a liver transplant did not change the mortality rate during follow-up with statistical significance (*P* = 0.674) (Fig. 3). Liver transplantation was performed on 24 of 163 WD patients with liver cirrhosis. Their mortality rate during follow-up was 4.2%, but it was 16.5% without liver transplantation. Nine of the 38 WD patients with liver failure received a liver transplant. Their mortality rate during follow-up was 11.1%, but it was 27.6% without liver transplantation (Table 5).

**Table 5:** Mortality rate during follow-up by liver transplantation in patients with liver failure or cirrhosis.

	Total no of patients	No of LT	Mortality rates	
			With LT	Without LT
Liver cirrhosis	163	24 (14.7%)	1 (4.2%)	23 (16.5%)
Liver failure	38	9 (23.7%)	1 (11.1%)	8 (27.6%)

LT, liver transplantation.

## Discussion

In our study, the average annual incidence rate of WD was 3.8 per million person-years. This is comparable to that reported in other recent population-based studies in Asian countries. The average annual incidence rate was 1.4 per million person-years in Hong Kong in 2017 and 2.7 per million person-years in Taiwan in 2010^11,13^. Some older studies reported much higher incidence rates, ranging from 17 per million person-years in Ireland in 1993 to 29 per million person-years in East Germany in 1979^14,15^. But the number of cases in those studies was 26 and 126, respectively. The variance in incidence is thus attributed to cohort size and sociodemographic characteristics.

The average annual prevalence of WD was 38.7 per million people. Some studies in European countries had identified a clinical prevalence of WD ranging from 12 to around 30 per million people^11–13,16^. That difference might reflect an actual increase in the number of patients because of an accumulation of patients who survive after a diagnosis of WD thanks to advances in diagnostic techniques, including genetic studies, and treatment methods. The point, that WD was diagnosed in a young age and had higher mortality in the elderly, could cause an accumulation of surviving patients after the diagnosis of WD. In our data, the annual prevalence of WD increased throughout the study period, from 28 per million people in 2010 to 48.1 per million people in 2016. Increasing prevalence was also confirmed in other recent population-based studies^11–13^. Therefore, we predict that the prevalence will continue to increase, even if there is no change in the incidence rate. The prevalence of a WD diagnosis was higher among young people than older people. Of the patients newly diagnosed with WD, 77.8% were younger than 40 years, and previous studies also show that most WD patients are diagnosed when they are younger than 40 years. In our study, 43.1% of patients were younger than 20 years old when they were diagnosed^11–13^. Therefore, young patients with idiopathic liver disease should be tested for WD.

Hepatic manifestations are common symptoms of WD. In this study, 74.1% of all WD patients had hepatic symptoms. The diagnostic age of liver disease in WD patients is similar to the diagnostic age of WD, indicating that liver disease can be a point for the diagnosis of WD. The connection could be more cogent with hepatitis, which developed in 68.6% of WD patients in our study. The incidence of hepatitis among our WD patients was similar to that in the recent Hong Kong population-based study and a large Austrian cohort study^11,17^. The age at which patients were diagnosed with liver disease was younger among men WD patients than women. This might be correlated with younger age at diagnosis with WD in men than women, considering the incidence of liver disease in all WD patients, even though the most common histologic finding of WD in childhood is steatosis. Several previous studies considered HCC in WD. The first case of HCC in a WD patient was reported in 1959^18^. In another previous study, the onset age of WD varied from 12 to 66, but the mean diagnostic age was 39^19^. In our study, 132 WD patients developed HCC, which was 9.9% of all WD patients, and the mean diagnostic age of HCC in WD patients was 33.7 ± 17. That was similar to the age reported in another study and much younger than the mean diagnostic age of HCC in the general population. The incidence of HCC in WD patients was lower than 3% in previous studies^11,20–23^. Among 1,278 WD patients who did not have HCC at diagnosis, 77 (6%) developed HCC during follow up. Considering 7 years from 2010 to 2016, the rate seemed not to be high. We could not identify individualized confirmation for viral hepatitis infection in WD patients, because of the limitations of big-data research. Further cohort study seems to be needed about this. Also, differences in the cohorts between studies might explain at least some of the difference in incidence. Nonetheless, our result could have clinical significance because our study used a larger population than previous reports. It is important that the mean diagnostic age of HCC in WD patients was younger than the mean diagnostic age of HCC in the general population. In addition, the difference between the mean diagnostic age of liver cirrhosis in WD patients and the mean diagnostic age of WD was only about 6 years. Thus, to prevent progression to advanced liver disease and avoid diagnosis delays for WD patients, young patients who present with hepatitis without any evident cause should be screened for WD.

Neurologic disease was diagnosed in 38.3% of WD patients in our study. Neurologic symptoms were previously reported in 40–50% of WD patients, and it usually developed in their twenties^24^. In our study, the mean diagnostic age of neurologic disease in WD patients was 31.5, but 68.6% (350 of 510) of those patients had visited medical institutes for neurologic symptoms presumed occurring before diagnosis of WD. In addition, 105 of the patients with tremor (56.5%) and 10 of the patients with chorea (58.8%) had been treated for neurologic symptoms without WD being considered as a causative factor. Psychiatric disease was observed in 45.1% of WD patients, and 71% (427 of 601) of those patients had visited medical institutes for psychiatric symptoms presumed occurring before diagnosis of WD. Demily et al. reported that copper metabolic abnormalities had been observed in 19% of patients hospitalized for psychiatric disease with no history of a WD diagnosis. Mutation in the *ATP7B* gene was found in 3.4% of those patients^25^. It has been reported that proper management of WD patients with psychiatric problems could improve their cognitive and behavioral symptoms and that early treatment for WD could prevent the onset of neurologic presentation^26,27^. Considering that many patients develop neuropsychiatric symptoms before they receive a WD diagnosis and that their age at diagnosis is young on average, it is necessary to check the liver function and consider a workup for WD when young patients without any organic brain lesions present with neuropsychiatric symptoms.

Renal disease was diagnosed in 4.4% of WD patients in our study. It was relatively rare and may be a mild manifestation. But considering the report that decoppering treatment may result in kidney impairment and that proteinuria was observed in 7% of WD patients treated with penicillamine^28^, more attention should be paid to renal function checks in WD patients. The most common cardiac manifestation was arrhythmia, observed in 13% of all WD patients, who were diagnosed in their 30 s on average. Considering the risk of atrial fibrillation was 29% higher in WD patients and electrocardiographic abnormalities presented in a third of WD patients in a previous study^28^, cardiac manifestations are not rare and must not be overlooked. Osteoporosis was diagnosed in 6.8% of WD patients in our study, at the age of 47.9 ± 17.5 years on average. Osteoporosis is often diagnosed at a relatively old age, and may include degenerative changes with age. This condition therefore requires a differential diagnosis with degenerative disease.

The annual mortality rate among WD patients during follow-up was 0.7% on average and increased with age. However, although the mortality rate among WD patients in recent studies of the period from 2005 to 2014 ranged from 1.8 to 21.1%, the tendency of increased mortality with age and low disease-related mortality overall was consistently identified^17,22,23,29^. The increase in mortality with age could be associated with the development of WD-associated diseases and the progression of liver disease. However, the aggravation of other underlying diseases might also have influenced the rising disease-related mortality rates among elderly WD patients. In this study, the cause of death could not be identified, and some patients might have died from causes unrelated to WD. Mortality among young patients diagnosed with WD was relatively low. Therefore, early diagnosis and appropriate management of WD could prevent disease progression and the development of complications, which could decrease the disease-related mortality rate by age.

The factors correlated with death were age, liver failure, and liver cirrhosis. Mortality during follow-up showed no significant relationship with medical treatment, sex, or liver transplantation. On the contrary, the medical treatment group tended to have a slightly higher mortality rate than the group that received no medical treatment. This is explained by the limitations of Big-data research. We used NHIS database, so medical treatment beyond insurance coverage is likely to have not been checked. Also, in our data, it was difficult to identify individual clinical factors such as liver function and other symptoms. An additional prospective study could better identify the clinical effects of medical treatment for WD.

Liver transplantation is the ultimate option for patients with impaired liver function. Previous studies showed survival rates after liver transplantation ranging from 62% at 12 months to 100% at 33 months^24,30–32^. In this study, the survival rates of patients who received liver transplantation were 100%, 95.8%, 95.8%, and 92.3% at 1, 2, 3, and 4 years, respectively. However, liver transplantation did not affect the mortality of WD patients in our study. Fifty-six of the WD patients in our study received liver transplants. Their mortality rate during follow-up was 5.4%, which was higher than the overall average. But considering that patients who received liver transplantation had a high MELD score or complications such as liver failure or HCC, it is not at all certain that liver transplantation in advanced WD is unhelpful. In our results, the mortality rate among patients with liver cirrhosis who received a liver transplant was 4.2%. But patients with liver cirrhosis who did not receive a liver transplant had a mortality rate during follow-up of 16.5%. Among WD patients with liver failure, those who received a liver transplantation also showed a lower mortality rate during follow-up than those who did not (11.1% vs. 27.6%). Although liver transplantation did not affect the overall mortality rate of WD patients during follow-up, it apparently did reduce mortality among WD patients with liver cirrhosis or liver failure.

This study had some limitations. First, the selection of WD patients was based only on the registration of KCD code E830, so errors in registration cannot be completely eliminated. Although WD is a rare disease and confirmative diagnosis was based on specific clinical studies, some patients who lacked evidence for confirmation might have been included. On the contrary, some patients with WD might not have been included because they could not meet the diagnostic criteria or died before confirmation. Second, diagnosis with the associated diseases was also based on the registration of disease-specific codes, so some patients might have been missed or misdiagnosed, and the time at which the code was entered (our diagnostic point) might have been later than the time at which symptoms appeared. Third, clinical manifestation is an important point in identifying the efficacy of medical treatment and disease progress. However, our study is based on big-data, so we could not analyze detailed clinical information for each patient. So even though liver transplantation patients showed lower mortality rates during follow-up than patients with liver cirrhosis or liver failure who did not receive a transplant, unidentified clinical differences could have caused errors in our analysis. Fourth, although associated diseases were identified among the patients diagnosed with WD using EDI codes, WD cannot be a crucial cause of all described symptoms. WD is only one of the highly suspected causes. Therefore, comparative study of the prevalence of associated symptoms between WD patients and non-WD patients is needed to confirm our results.

In conclusion, this large population-based study has shown the incidence rates and prevalence of WD in South Korea from 2010 to 2016. The prevalence of WD increased annually. Age, liver cirrhosis, and liver failure correlated with mortality during follow-up in WD patients. Neurologic and psychiatric diseases developed in a high proportion of WD patients, and about 70% of them presented with neuropsychiatric symptoms presumed occurring before diagnosis of WD. they were diagnosed with WD. Therefore, WD needs to be considered in patients without organic brain lesions who have neurologic or psychiatric symptoms at a young age. Further research is needed on HCC and medical treatment in WD patients.
